# Supplementary material for: Attosecond optoelectronic field measurement in solids
Source: Nat Commun. 2020 Jan 22;11:430. doi: 10.1038/s41467-019-14268-x (PMC6976600; doi:10.1038/s41467-019-14268-x)
Supplement: Supplementary file 1 — Supplementary Information [file 41467_2019_14268_MOESM1_ESM.pdf]

Supplementary Information

Attosecond optoelectronic field measurement in solids

Sederberg *et al.*

Supplementary Note 1 – Signal processing

The raw waveform data are processed with a bandpass filter of supergaussian form in the frequency domain,

$$S(f) = e^{-\left(\frac{f-f_0}{2\sigma}\right)^8}. \quad (1)$$

The parameters of the filters applied in the relevant figures are listed in Supplementary Table 1.

Supplementary Note 2 – EOS detection and data processing

The detailed measurement of the apparent time delay of the $G(t)$ function of NPS is performed using electro-optic sampling (EOS) of a 1.8-μm field. While in principle, attosecond streaking would also be sensitive to such an offset, it relies on a different pair of pulses at very different photon energies, and would thus not permit a direct comparison of the gating in this case.

The EOS detection system is quite similar to that described in.[1] The EOS crystal used is a type II BBO (such that the probe pulse and detected field have orthogonal polarization), with a phase matching angle (θ) of 50.0 degrees, and thickness of 12.4 μm, as measured with a white light interferometer. The detector response function is calculated using the known optical sampling pulse, using the nonlinear wave equation[1]

$$\frac{\partial E_\omega(z)}{\partial z} = -ik(\omega)_\omega E_\omega(z) - \frac{i\omega}{2n(\omega)\epsilon_o c} P_\omega^{(NL)}(z), \quad (2)$$

which describes the evolution of each spectral component of the light $E_\omega(z)$ as it propagates in the crystal, with wavevector $k(\omega)$, refractive index $n(\omega)$ and corresponding spectral component (at frequency ω) of the nonlinear polarization $P_\omega^{(NL)}(z)$ under the slowly evolving wave approximation. The nonlinear polarization is calculated using the full tensor of the second order nonlinear response for the light on both axes of the crystal, and the third order response using only the self-phase modulation (SPM) and cross-phase modulation terms (XPM), taking its tensor nature into account.[2] Under the experimental conditions, the intensity in the EOS crystal is not sufficient for a significant contribution to the signal from SPM or XPM, which produce a maximal timing shift of 25 attoseconds according to the simulations of nonlinear propagation.

The response function $G(t)$ for the EOS process is obtained by numerically propagating the measured sampling pulse, and a broadband infrared test waveform through the crystal under varying time delays, analyzing the polarization state of the resulting probe light and calculating the resulting signal on the balanced detector based on the known response curve of the photodiodes and transmission

of the spectral filter. $G(t)$ is then obtained by inverse Fourier transformation of the ratio of the complex spectrum of the simulated signal to the complex spectrum of the known test waveform, with a bandpass filter applied to include only the near infrared spectral region. This calculated $G(t)$ is then deconvolved from the measured waveform to correct the spectral phases and amplitudes of the EOS signal to produce the electric field at the input facet of the nonlinear medium.

Supplementary Note 3 – Influence of screening and momentum relaxation

As bulk dielectrics have relatively large dielectric constants and the free-carrier densities injected by intense laser fields are significant (on the order of 10^{18} cm^{-3} and above), the form of the electric field incident on the material and the field inside the material will substantially differ from each other.

Each component of the field inside the material may be approximated by

$$E_{\mathrm{trans}} = E_{\mathrm{inc}} - \frac{E_{\mathrm{inc}} P/\epsilon_0}{4E_{\mathrm{inc}} + P/\epsilon_0}\,, \tag{3}$$

where E_{trans} is the field inside the surface of the material transmitted through the surface, E_{inc} is the incident field, and P is the polarization induced by E_{trans}. (This expression simply yields the Fresnel equations for $P = \chi E_{\mathrm{trans}}$ under the boundary condition $E_{\mathrm{trans}} = E_{\mathrm{refl}} + E_{\mathrm{inc}}$ for the reflected field E_{refl}.) We avoid numerical instabilities that appear when $P \approx -4\epsilon_0 E_{\mathrm{inc}}$ by approximating the RHS of supplementary equation (3) with the first two terms of its Taylor series expansion in P. While the polarization resulting from the linear response of the material can be simply obtained by convolution with a response function $\chi(t)$, the polarization due to the laser-induced charge carriers both depends on and influences the transmitted field and the two must be solved self-consistently.

We use the classical equation of motion for the current of free carriers $\mathbf{J}_e(t)$ driven by the local transmitted field $\mathbf{E}_{\mathrm{trans}}(t)$,

$$\dot{\mathbf{J}}_e(t) = -N(t)\mathbf{E}_{\mathrm{trans}}(t)/m^* - \gamma \mathbf{J}_e(t)\,. \tag{4}$$

The field present in the sample in our case is $\mathbf{E}_{\mathrm{trans}} = E_{\mathrm{d}}\hat{\mathbf{x}} + E_{\mathrm{i}}\hat{\mathbf{y}}$, $N(t) = \int_{-\infty}^{t} dt' w(t')$ is the free carrier density, m^* is the reduced effective mass of an electron-hole pair and γ describes the rate of momentum relaxation. For simplicity, the carrier injection rate $w(t)$ is approximated as $w(t) = 10^9 E_{\mathrm{i}}^8(t)$ a.u. in the following analysis, consistent with the energy transfer dynamics observed in the time dependent density functional theory (TDDFT) calculations discussed in the manuscript. The total current is given by $\mathbf{J} = \mathbf{J}_e + \mathbf{J}_b$ where $\mathbf{J}_b$ is the response of the bound electrons, including the linear refractive index and the nonlinear absorption response. The induced polarization follows as

$\mathbf{P}(t) = \int_{-\infty}^{t} dt' \mathbf{J}(t')$. The signal from the electrodes will then be proportional to the DC component of $\mathbf{P}(t)$.

The presence of a small density of free carriers in a dielectric leads to a reduction in its refractive index, as the polarizations induced by the free and bound carriers oppose one another, reducing the overall polarizability of the material. As this happens in a time-dependent manner during the course of the light-matter interaction, it shifts the relative weight of the portions of the electric field that come before and after the charge injection, which in turn influences the apparent relative timing of the field with respect to the injection of charge carriers. As the screening polarization is sensitive to both carrier density (controllable through field strength) and momentum relaxation time, understanding the influence of these factors is important for understanding the attosecond-scale control of currents with light fields.

Supplementary Figure 1 shows how the induced charge carrier density changes the signal measured as a function of time delay between the injection and drive pulses, for a momentum relaxation rate γ = 0.005 a.u. (corresponding to ca. 30 fs relaxation time), and with a relaxation rate γ = 0.5 a.u. (corresponding to ca. 300 as relaxation time) in Supplementary Figure 2. The form of the trace is essentially independent of the screening effects and primarily exhibits only a temporal shift with carrier density.

To look more specifically at the effect of momentum relaxation on the waveform retrieved through the measurement, the same simulation is performed with fixed electric field strength (0.5 VÅ^{-1}) and varying relaxation time in Supplementary Figure 3. The form of the signal waveform is almost completely independent of momentum relaxation. Overall, because the net force on the carriers is always the integral of the electric field after they are injected and the momentum relaxation presents a linear opposing force, the dependence on this parameter is primarily a simple rescaling of the magnitude of the current. This apparent lack of influence over the shape of the waveform is beneficial for field sampling applications, as the true relaxation dynamics are not exactly known. This weak dependence on momentum relaxation time is in contrast to the dependence on the rate of recombination—when it is shorter than the period of the electromagnetic field, the signal is proportional to the electric field, rather than the vector potential. However, this timescale is significantly longer than momentum relaxation, typically longer than picoseconds, and observable via photoemission experiments.

Finally, since the carrier density affects the delay between the vector potential and the sampled waveform, it is important to take its spatial dependence into account when comparing simulations calculated for a single field strength vs the experiment by spatial averaging. This is done by assuming a Gaussian focal spot and performing a weighted average over the simulated intensities. As a result,

the overall delay is reduced vs. the single-intensity calculations as shown in Supplementary Figure 4.

Supplementary Note 4 – Time-dependent density functional theory

We have performed *ab-initio* simulations of energy transfer into the bulk dielectric in order to test and to gauge simplified phenomenological models for the energy that allow to identify different orders of nonlinearity in the response. To this end, we have performed time-dependent density functional theory (TDDFT) calculations using the SALMON package solving the time-dependent Kohn-Sham equations in velocity gauge in real space and real time.[3] The Kohn-Sham orbitals are discretized on a Cartesian grid in real and reciprocal spaces. Core electrons are frozen in norm-conserving pseudopotentials. To account for the correct band gap frequently underestimated within DFT we employ the adiabatic Tran-Blaha modified Becke-Johnson exchange correlation functional.[4] Linear response properties of SiO_2 are reasonably well reproduced.[5]

The total excitation energies of the crystal at the conclusion of the pulse are plotted as a function of the field strength in Supplementary Figure 5. The energy is proportional to E_{i}^{8}, as shown by the fitted line on the log-log plot. This suggests that the time-dependent energy transfer, once transient terms are removed, might also follow a simple expression.

The calculated time-dependent energy transfer is shown in Supplementary Figure 6, the full energy exchange with the laser in Supplementary Figure 6(b), which also includes the transient energy exchange due to the linear and nonlinear refractive indices of the material. The energy transfer functions at peak field strengths ranging from 0.4 to 2.0 VÅ^{-1} are fitted to the ansatz

$$W(t) = E_0^2 a(t) + E_0^4 b(t) + E_0^n c(t), \tag{5}$$

where $W(t)$ is the work done on the system, E_0 is the peak field strength, $a(t)$ describes the energy exchange due to the linear response of the system, $b(t)$ describes the energy exchange due to the third-order nonlinear response, and $c(t)$ describes the energy exchange due to the higher-order nonlinear response. We find very good agreement with all data sets for $n = 8$. The extracted energy transfer after subtracting the transient contributions originating from the first- and third-order responses agrees well with a rate of energy deposition proportional to $E^8(t)$, i.e. a highly non-linear function of the time-dependent field, but with a small delay of $\approx$ 75 as.

The identification of the microscopic mechanism for this time delay predicted by TDDFT for strong-field dynamics in condensed matter is still an open question. We note, however, that for strong-field ionization in argon similar time delays have been experimentally observed in the above-threshold ion-

ization (ATI) regime.[6] Also, recent *ab initio* simulations of strong-field ionization of helium indicate that two-photon ionization is considerably delayed relative to one-photon ionization when the same final energy in the continuum is reached. This suggests time delays when high-order photon absorption, as possible in the present case, are involved.

Supplementary Note 5 – Spectral response

The spectral response of the method may be obtained from the results of the TDDFT simulations, simply by Fourier transformation of the energy transfer after the removal of the lower-order terms. As can be seen in Supplementary Figure 7, the cos pulse leads to a relatively flat phase response up to the cut-off frequency of 1200 THz.

The structure of the spectral response can be well understood within the ansatz employed for the gate function $G(t) \approx E^8(t)$. The measured signal is the convolution of this function with the time-integrated electric field. In the spectral domain, $\tilde{G}(\omega)$ and $\tilde{A}(\omega)$ are multiplied. Distortions that arise from a finite duration of $G(t)$ can be understood easily in the frequency domain, and, to a degree, corrected if the injection field is known by dividing $\tilde{S}(\omega)$ by the calculated $\tilde{G}(\omega)$ (i.e. deconvolution), with appropriate band-pass filtering to avoid the strong increase of noise in areas where $\tilde{G}(\omega)$ approaches zero.

In the limit of extremely short laser pulses, this approximate spectral response of the measurement approaches a constant value, $\tilde{G}(\omega) = \text{const}$. For more readily obtainable laser pulses of few-cycle duration, one cannot generally assume that the injection is always confined to a single half cycle. The simple approximation of $G(t)$ makes it possible to derive the spectral response and pulse duration requirements of the NPS measurement when the injection pulse has a known shape. For an injection field described by $E_\mathrm{i}(t) = \mathrm{Re}[\tilde{F}_\mathrm{i}(t)\exp(-\mathrm{i}\omega_\mathrm{L}t + i\phi_\mathrm{CE})]$ and a gate described by $G_{2n}(t) \approx E^{2n}(t)$, the spectral response is

$$\tilde{G}_{2n}(\omega) = \sum_{k=-n}^{n} C^{2n}_{n-k}\tilde{F}_{2n}(\omega - 2k\omega_\mathrm{L})e^{i2k\phi_\mathrm{CE}} \tag{6}$$

where $\tilde{F}_{2n}(\omega) = \int_{-\infty}^{\infty} dt e^{-i\omega t}\tilde{F}^{2n}(t)$ is the Fourier transform of the complex envelope raised to the appropriate power, and C^{2n}_{n-k} are the binomial coefficients. The spectral response is a series of replicas of the spectrum of the self-gated envelope, repeated with central frequencies $\omega = 0$, $\omega = 2\omega_\mathrm{L}$, $\omega = 4\omega_\mathrm{L}$, and so on, up to a maximum determined by $2n\omega_\mathrm{L}$, weighted by the binomial coefficients. Only for sufficiently broadband pulses will the spectra of the replicas at $\omega = 0$ and $\omega = 2\omega_\mathrm{L}$ overlap, allowing for gapless sensitivity for all frequencies in the observed range, and only for $\phi_\mathrm{CE} = N\pi$

(for integer N) will they have maximal constructive interference. For $\phi_{\mathrm{CE}} = N\pi + \pi/2$, they will interfere destructively, producing the minimum observed in the measurements (Figure 3) near the carrier frequency ω_{L}. This is also the reason for the π jump in the spectral phase of the response observed in the TDDFT simulations with a sin-like pulse in Supplementary Figure 7.

The response in the spectral range near $\omega = \omega_{\mathrm{L}}$ is strongly dependent on having an extremely short injection field, and will rapidly drop in amplitude relative to the rest of the detected spectrum as the pulse duration is increased. By assuming a Gaussian pulse shape, explicit limits on the duration of the pulse can be imposed. When $\tilde{F}_{\mathrm{i}}(t) = e^{-\frac{t^2}{2\sigma^2}}$

$$\tilde{G}_{2n,\mathrm{Gaussian}}(\omega) = \frac{\sigma\sqrt{\pi}}{\sqrt{n}} \sum_{k=-n}^{n} C_{n-k}^{2n} e^{-\frac{\sigma^2}{4n}(\omega - 2k\omega_{\mathrm{L}})^2 - i2k\phi_{\mathrm{CE}}}. \tag{7}$$

The loss of spectral amplitude near the carrier frequency when $\phi_{\mathrm{CE}} = 0$ can be parameterized simply through the ratio between the amplitudes at ω_{L} and $2\omega_{\mathrm{L}}$, the relative depth of the minimum in the spectrum:

$$\frac{\tilde{G}_{2n,\mathrm{Gaussian}}(\omega_{\mathrm{L}})}{\tilde{G}_{2n,\mathrm{Gaussian}}(2\omega_{\mathrm{L}})} \approx \frac{C_n^{2n} + C_{n-1}^{2n}}{C_{n-1}^{2n}} e^{-\frac{\sigma^2}{4n}\omega_{\mathrm{L}}^2} \tag{8}$$

where the approximation has been made that only the terms of the sum for $k = 0$ and $k = 1$ contribute (i.e. the replicas of the pulse spectrum centered near $\omega = 0$ and $\omega = 2\omega_{\mathrm{L}}$). This amplitude will fall by $1/e$ for

$$\sigma_{\max} = \frac{2\sqrt{n\left[\ln\left(\frac{C_n^{2n} + C_{n-1}^{2n}}{C_{n-1}^{2n}}\right) + 1\right]}}{\omega_{\mathrm{L}}}. \tag{9}$$

This value can be viewed as a guideline for the maximum applicable pulse duration, assuming that the pulse's complex envelope can be measured accurately via FROG or similar techniques to correct the spectral weights, beyond which the deep hole in the spectrum could be difficult to correct reliably or with adequate signal-to-noise ratio. For example, for $n = 4$, this corresponds to an intensity full-width-at-half-maximum duration of 3.8 fs at 800 nm carrier wavelength.

Supplementary Note 6 – Harmonic distortions and signal-to-noise ratio

At very low strengths of the driving field, the signal-to-noise ratio (SNR) of the measurement increases as the drive field intensity increases, since the main source of noise is electronic background in the measurement. Above a certain limit, however, the total noise in the measurement will be dominated by the noise contained in the signal itself. At this point, increasing the strength of the driving field does not significantly increase the signal-to-noise ratio of the measurement, at least in the temporal

region close to the field maximum. Increasing SNR at this value is dependent on improving the pulse-to-pulse stability of the driving laser system.

At yet higher values of E_d, such that the assumption that $E_\mathrm{d} \ll E_\mathrm{i}$ is no longer valid, E_d will significantly participate in the carrier injection process, resulting in distortions of the measured signal first visible as harmonic distortions. Both of these properties of the measurement are most easily observed through the recorded spectrum over a range of strengths of E_d, as presented for two different driving wavelengths (1.8 μm and 750 nm, which provide the largest dynamic range and highest available intensity, respectively) in Supplementary Figure 8 for an injection field strength of 1.7 VÅ^{-1}. The onset of these distortions in this set of measurements was when E_d approached approximately 1/2 of E_i.

It can be seen that the measurement enters the range of signal-fluctuation-limited SNR near 0.3 VÅ^{-1}. In Supplementary Figure 8(b), the "signal" line (208 THz) and "noise" lines (all other frequencies outside the signal bandwidth) increase with approximately the same slope above this value. The SNR is plotted in Supplementary Figure 9, as the ratio between the signal line and average of the noise lines - although it is subject to significant scatter due to the noise term being in the denominator. In the low-driving-field range, the SNR increases with field, and at the higher-field range reaches a plateau.

The combination of a limiting field strength above which harmonic distortions occur, and decreasing benefit in terms of signal-to-noise ratio define an optimal working range in terms of the peak driving field strength. Under our experimental conditions, this was from $\approx$ 0.2-0.8 VÅ^{-1}.

Supplementary Note 7 – Comparison of measured spectra

The spectra obtained through Fourier transformation of the waveform data presented in Figure 5 of the main text are shown in Supplementary Figure 10 for those wavelengths for which a grating spectrometer were available. The spectrometers used were an OceanOptics NIR512 for the 1.8-μm pulse and OceanOptics Maya for the three other traces. The spectra are in approximate agreement, although differences may arise from differences in how the measurements take place: the NPS spectra are sampled from a small region of the focused beam, while the spectrometer averages over the spectrum of the whole beam. Thus, spatio-spectral distortions that cause the spectra of different locations in the focus to differ will result in a disagreement between the two measurements.

One may note several features in the spectrum of the driving laser pulse in Supplementary Figure 10(b). The NPS measurement reproduces (within the limits of the spectral resolution) the structure of the spectrum, with the exception of the region above 600 THz (500 nm). This is the limit of the

working range of the chirped mirrors used to compress the light. Above this frequency, the reflectivity does not drop to zero, but the phase of the light varies strongly. As a result, the limited range of time delays in the attosecond streaking and NPS measurements does not fully account for the power in this region, as a significant portion of the energy is outside of the measurement window, with the in-window energy being dependent on the higher-order spectral phase.

Supplementary Note 8 – NPS and EOS comparison

The waveforms shown in Figure 4 are zoomed into the maximum of the envelope of the electric field, where the most reliable timing information exists. Supplementary Figure 11 shows a wider view of the pulses measured by the two detection techniques. The agreement between the waveforms is quantitatively better than the comparison with attosecond streaking, but there are still differences visible in the traces at the rising and falling edges of the pulse. This is due to the slightly different effective bandwidths of the measurements, which, in the case of a compressed pulse, lead to differences in the waveform proportional to the gradient of its envelope. For this reason, the timing comparison is done using the cycle of the waveform at the maximum of the envelope, where the time derivative is minimized.

Supplementary Note 9 – Detection limits

It is useful to know how much energy must be contained in a pulse before NPS can detect it. We show in Supplementary Figure 12 that even pulses on the nJ energy scale are detectable with NPS (injection pulse energy 7 μJ), through the observation of the waveform of a near-infrared (1400 nm) pulse generated with difference frequency generation, containing 1.4 nJ, attenuated with a pair of wiregrid polarizers. We compare with a pulse with 1000 times this intensity containing 1.9 μJ.

Supplementary Note 10 – Detection and modulation methods

In the measurement, a lock-in amplifier is used to detect the signal induced by the driving field. This requires the modulation at a fixed frequency. We can employ two methods of modulation for some of the measurements: either amplitude modulation by an optical chopper, or phase modulation by alternating the carrier-envelope-phase of adjacent pulses in the pulse train. The chopper can be employed for all nonlinear processes, but, for example, difference frequency generation cancels out the modulation placed on the input pulse and second harmonic generation will lead to a factor of two increase in the phase change. Data taken for the 750 nm pulse as driving field for consecutive measurements from both methods are shown in Supplementary Figure 13. The waveforms exhibit

similar characteristics, although they have slightly different amplitudes. The decay of the apparent persistent dipole induced by the laser fields in the sample is not fully understood at this point. The full dynamics of how the laser-induced dipole decays on the presumed nanosecond timescale, possibly under residual influence from pulse to pulse, may have a significant effect on the magnitude of the observed current, which is a topic worth exploring in future investigations.

Supplementary Note 11 – Measurement of phase accumulation in fused silica

As broadband pulses propagate through a medium, their spectral content becomes dispersed. Measurement of the spectral phase accumulated in a known material provides additional confirmation of the accuracy of the measured waveforms. Broadband spectral content is obtained by passing the 750 nm pulse through a BBO frequency doubling crystal. The resulting waveform is recorded and shown in Supplementary Figure 14a.

Fused silica substrates of varying thickness are inserted into the beam path and the transmitted dispersed waveforms are measured. The waveform transmitted through a $L = 0.5$ mm thick fused silica substrate is shown in Supplementary Figure 14b. Subtracting the spectral phase of the reference waveform and the linear phase from the temporal offset of the wave from that of the dispersed waveform provides the spectral phase accumulated in the substrate , as shown in Supplementary Figure 14c. Using refractive index data obtained from the Sellmeier equation[7] for fused silica:

$$n^2 - 1 = \frac{0.6962\lambda^2}{\lambda^2 - 0.06840^2} + \frac{0.4079\lambda^2}{\lambda^2 - 0.1162^2} + \frac{0.8975\lambda^2}{\lambda^2 - 9.8962^2} \tag{10}$$

we analytically calculate the expected spectral phase accumulation in the substrate, with no free parameters. The result of the calculation is shown in Supplementary Figure 14c, and demonstrates good agreement in the spectral region spanning from 300 THz to 725 THz. The same procedure is performed for a $L = 1$ mm thick fused silica substrate, plotted in Supplementary Figure 14d,e, and for a $L = 2.5$ mm thick fused silica substrate, presented in Supplementary Figure 14f,g.

Figure	element	f_0 (THz)	σ (THz)
2	NPS	600	273
4	EOS and NPS	170	38
5	275-330 nm	960	130
5	375-440 nm	560	170
5	460-1035 nm	460	130
5	1050-2850 nm	195	100
5	3000-5300 nm	80	40

Supplementary Table 1: Bandpass filters used in the processing of raw data. These are the parameters used in supplementary equation 1 for the corresponding figures in the main text.

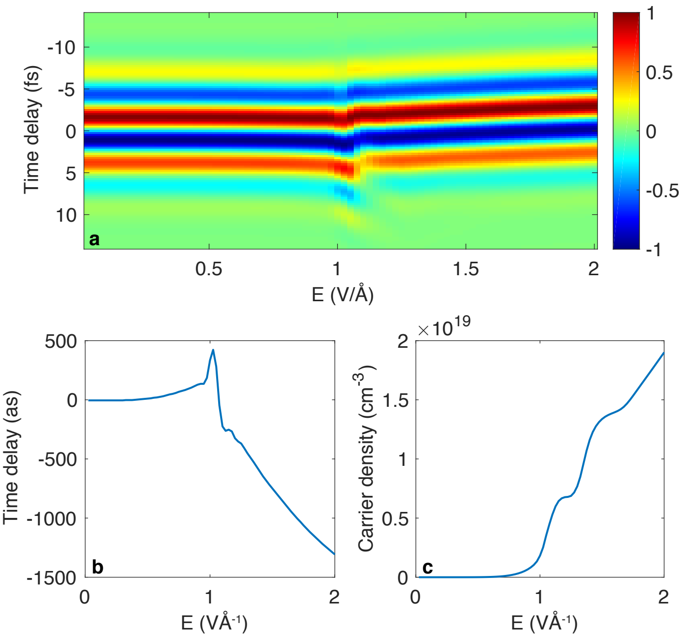

Supplementary Figure 1: Influence of injection field strength on signal, 30 fs momentum relaxation. In this case, γ = 0.005, corresponding to a relaxation time of $\approx$ 30 fs. a. Normalized induced signal as a function of time delay between injection and drive fields and the field strength. b. Time delay of the measured waveform relative to the vector potential of the drive field as a function of peak electric field strength. c. Final carrier density as a function of the peak electric field for $\gamma = 0.005$.

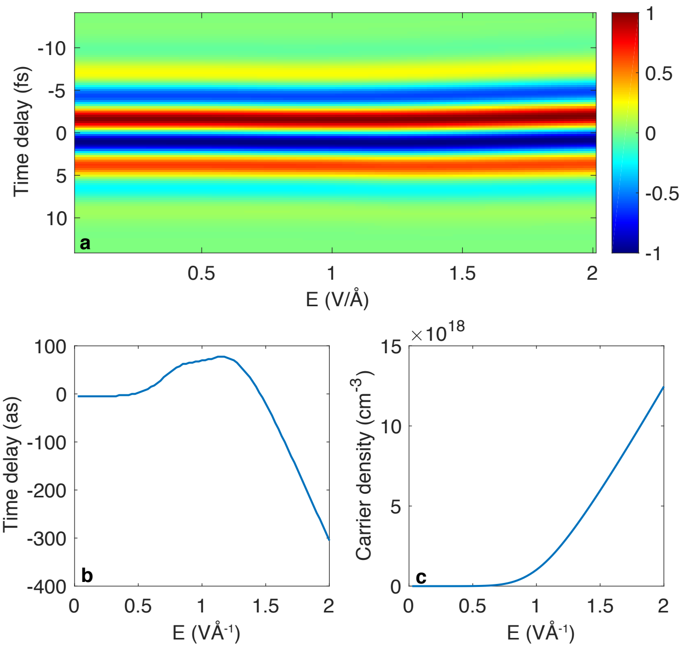

Supplementary Figure 2: Influence of injection field strength on signal, 0.3 fs momentum relaxation. In this case, γ = 0.5, corresponding to a hypothetical relaxation time of $\approx$ 300 as. Even such extremely fast relaxation rates result in a comparable form of the induced signal vs. time delay. a. Normalized induced signal as a function of time delay between injection and drive fields and of the field strength. b. Time delay of the measured waveform relative to the vector potential of the drive field as a function of peak electric field strength. c. Final carrier density as a function of field strength.

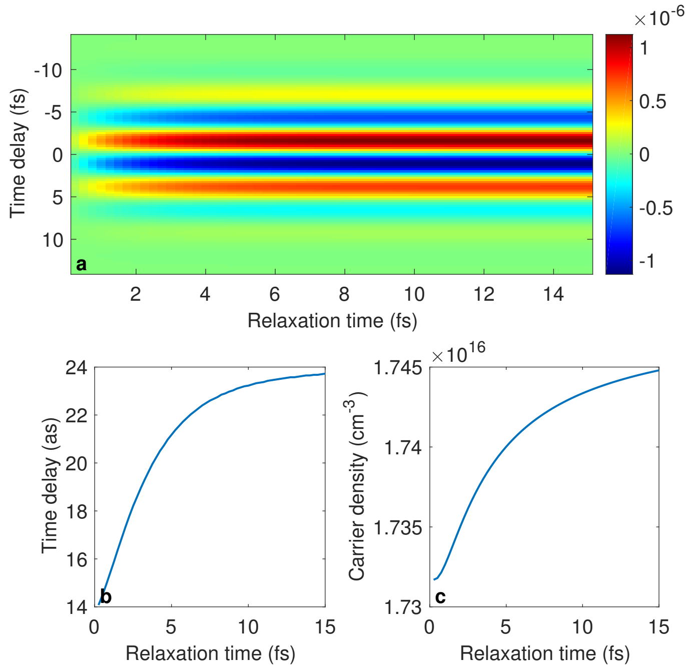

Supplementary Figure 3: Influence of momentum relaxation time on signal, field strength 0.5 VÅ^{-1}. a. Induced signal as a function of time delay between injection and drive fields and the momentum relaxation time. b. Time delay of the measured waveform relative to the vector potential of the drive field as a function of relaxation time. c. Final carrier density as a function of the momentum relaxation time.

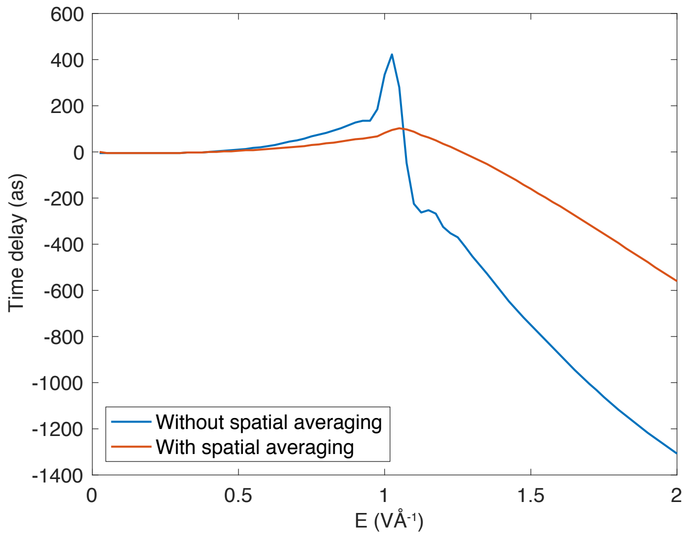

Supplementary Figure 4: Effect of spatial averaging. There is a significant influence of the nonuniform electric field strength over a Gaussian focus on the apparent time delay between vector potential and induced signal.

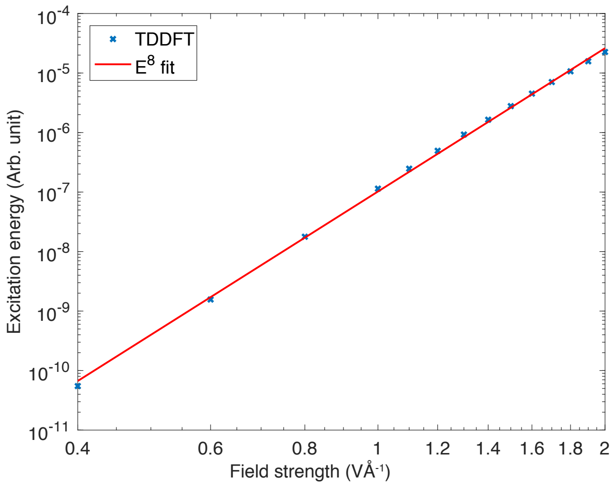

Supplementary Figure 5: TDDFT energy scaling. The scaling of the energy deposited into the unit cell at the conclusion of the laser pulse as calculated with TDDFT is well approximated as being proportional to the 8th power of the peak electric field strength.

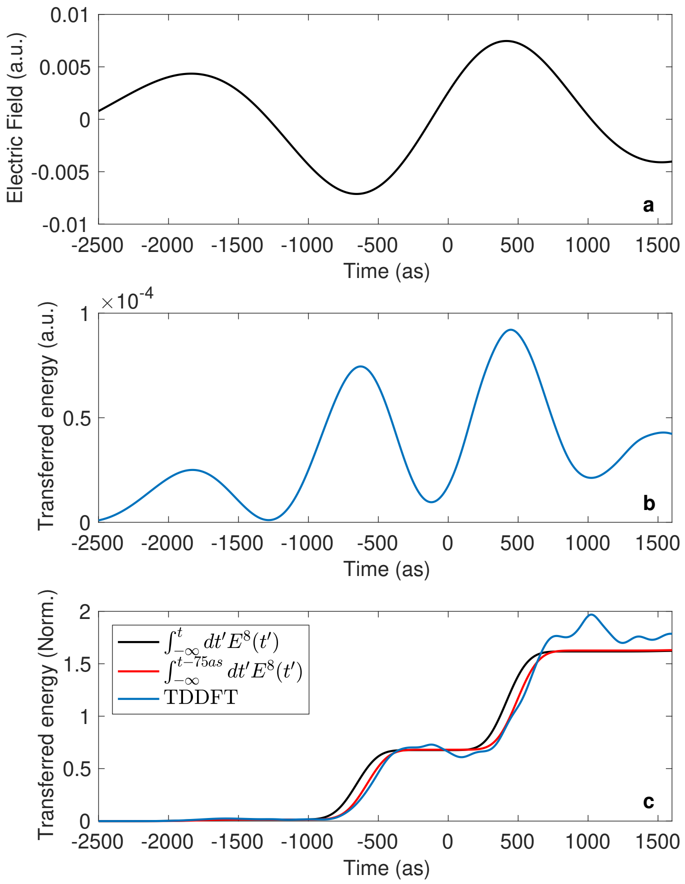

Supplementary Figure 6: Comparison of simple models of the transferred energy to TDDFT. The lower-order response is removed, allowing simplified models of the nonlinear response to be compared. **a** Time dependence of applied field near the peak of the envelope, where carriers are generated. **b** Energy transfer to the dielectric as calculated by TDDFT with a peak electric field of 2 VÅ^{-1}. **c** Energy transfer from the TDDFT calculation after removing the transient lower-order contributions compared with simple power-law models. The energy dynamics are well approximated by an energy deposition rate proportional to $E_{\mathrm{i}}^{8}(t)$, with a 75 as delay.

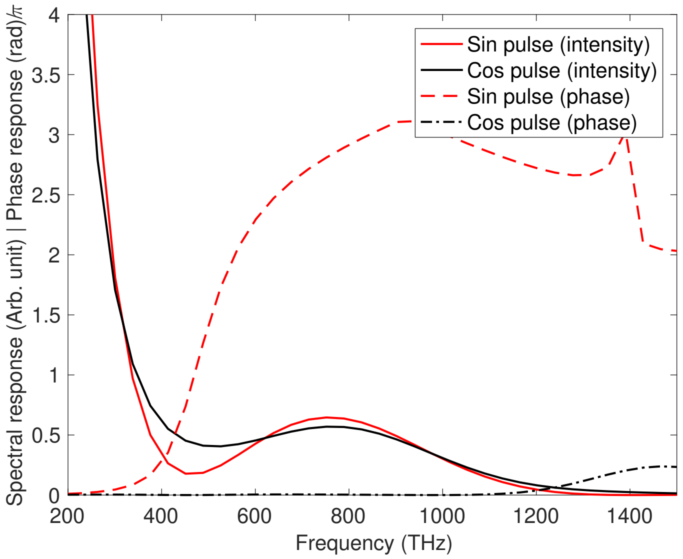

Supplementary Figure 7: TDDFT spectral response. The spectral intensity and phase responses of the detector are obtained via the TDDFT simulations, where Sin and Cos pulse correspond to the electric waveforms consisting of two approximately equal field extrema, or one single field extremum respectively.

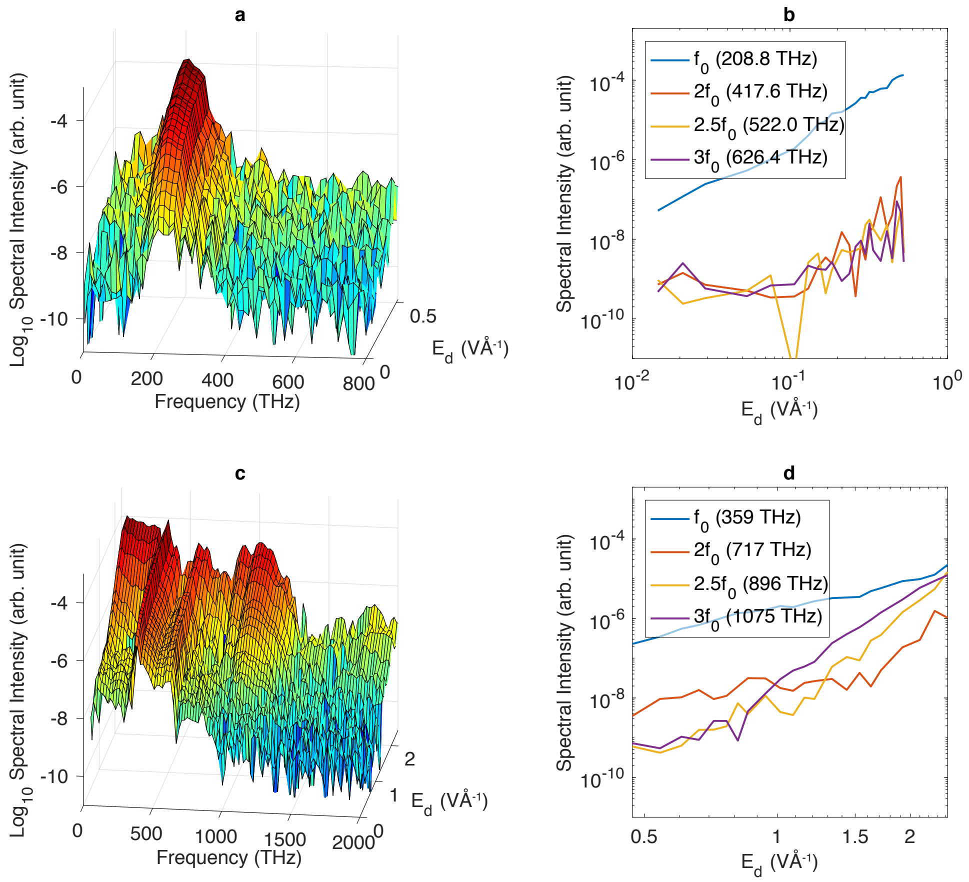

Supplementary Figure 8: Emergence of signal-induced noise and harmonic distortions. Harmonic distortions may appear at higher strengths of the driving field. **a** Spectrum of the signal with 1.8-μm driving field its intensity is increased. No harmonic distortions appear above the noise floor, but the average noise floor rises, indicating a significant contribution from signal fluctuations. **b** Lineouts of part **a** corresponding to the peak frequency of the driving field, its second and third harmonics, and the frequency equidistant from these harmonics. **c** Spectrum of the signal with 750 nm driving field in the high intensity range. Once the field exceeds $\approx$ 0.9 VÅ^{-1}, harmonic distortions appear, primarily at the third harmonic. **d** Lineouts of part **c** corresponding to the peak frequency of the driving field, its second and third harmonics, and the frequency equidistant from these harmonics.

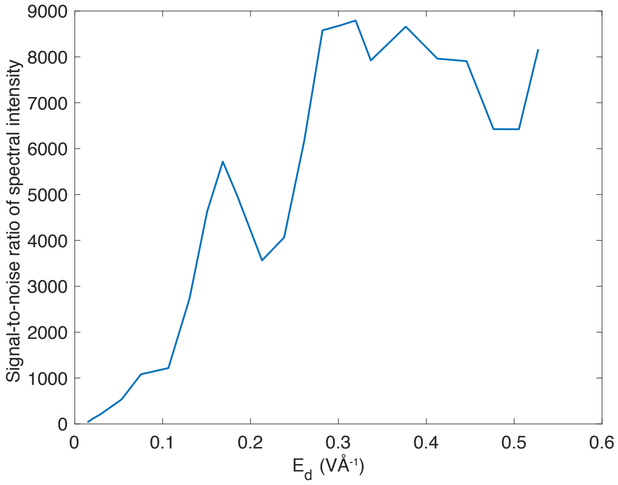

Supplementary Figure 9: Dependence of signal-to-noise ratio on driving field strength. The SNR increases at low field strengths of the 1.8-μm driving field, and reaches a plateau as it becomes dominated by noise from signal fluctuations.

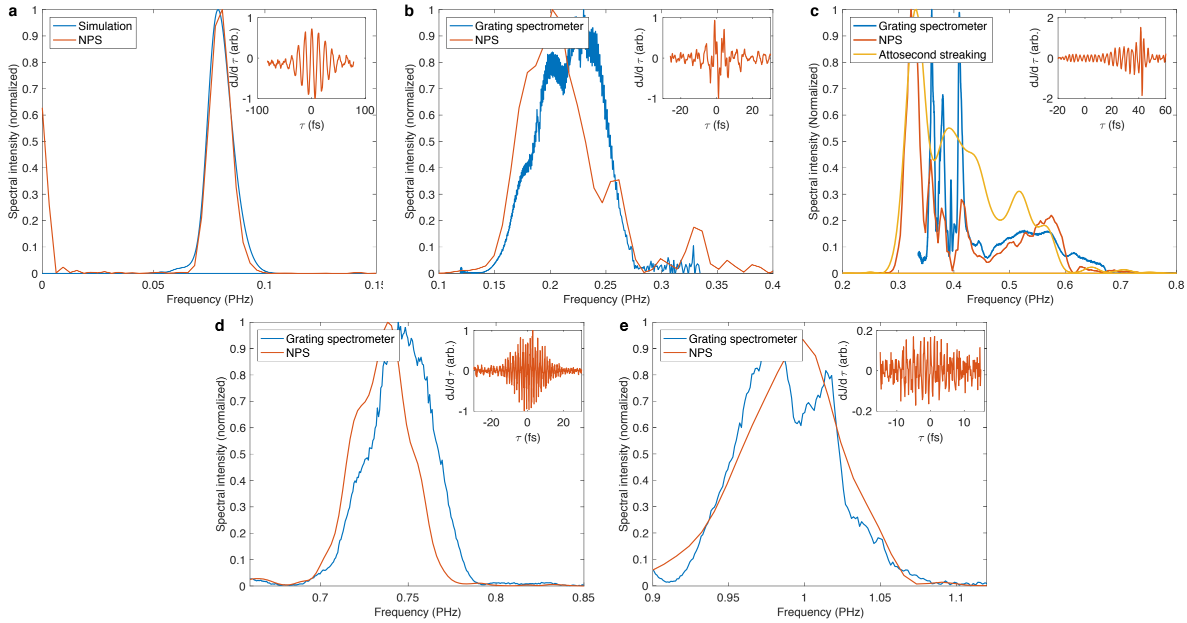

Supplementary Figure 10: Comparison of the the spectra of NPS measurements with references. The insets in all images correspond to the measured data with no bandpass filters applied. **a** difference-frequency generation (DFG) in 1 mm $LiNbO_3$, compared with the simulated spectrum using the propagation equation, supplementary equation (2). **b** DFG in BBO compared with grating spectrometer. **c** Unconverted NIR-visible pulse compared with grating spectrometer and attosecond streaking. **d** sum-frequency generation (SFG) in BBO, phase-matched to 400 nm, compared with grating spectrometer. **e** SFG in BBO, phase-matched to 300 nm, compared with grating spectrometer.

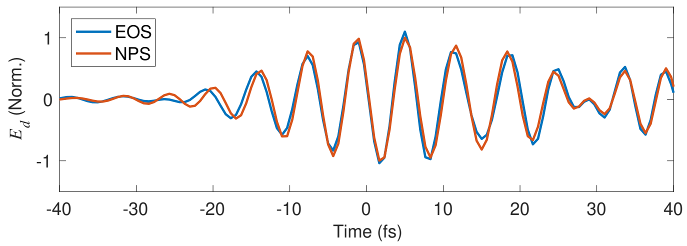

Supplementary Figure 11: Comparison of the waveforms obtained via EOS and NPS. These are plotted over a wider time-scale to show the range of the intensity envelope rather than the central cycle.

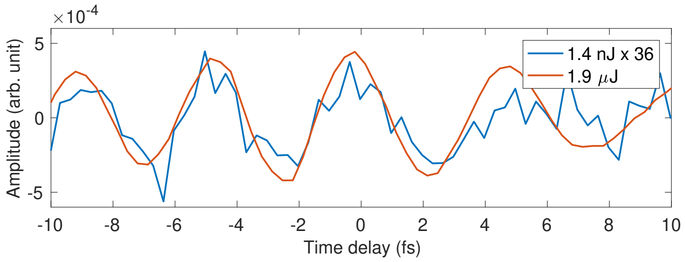

Supplementary Figure 12: NPS measurements, with the driving field containing 1.4 nJ and 1.9 μJ. Despite the factor of 1000 reduction in intensity, the field remains detectable down to the nJ scale. The low-intensity pulse is multiplied by the square root of the ratio of the high and low pulse energies (the approximate ratio of electric field strength), for comparison. No bandpass filters have been applied.

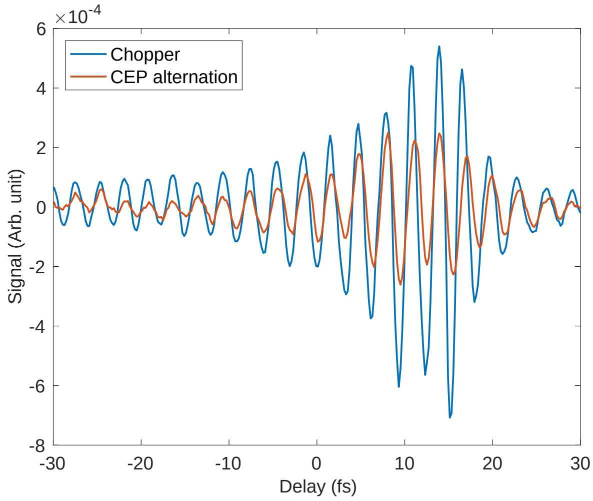

Supplementary Figure 13: Signals recorded with lock-in amplifier and CEP modulation or chopper. The measured waveform should be independent of the modulation technique, but differences in amplitude can indicate the persistence of the dipole formed in the material.

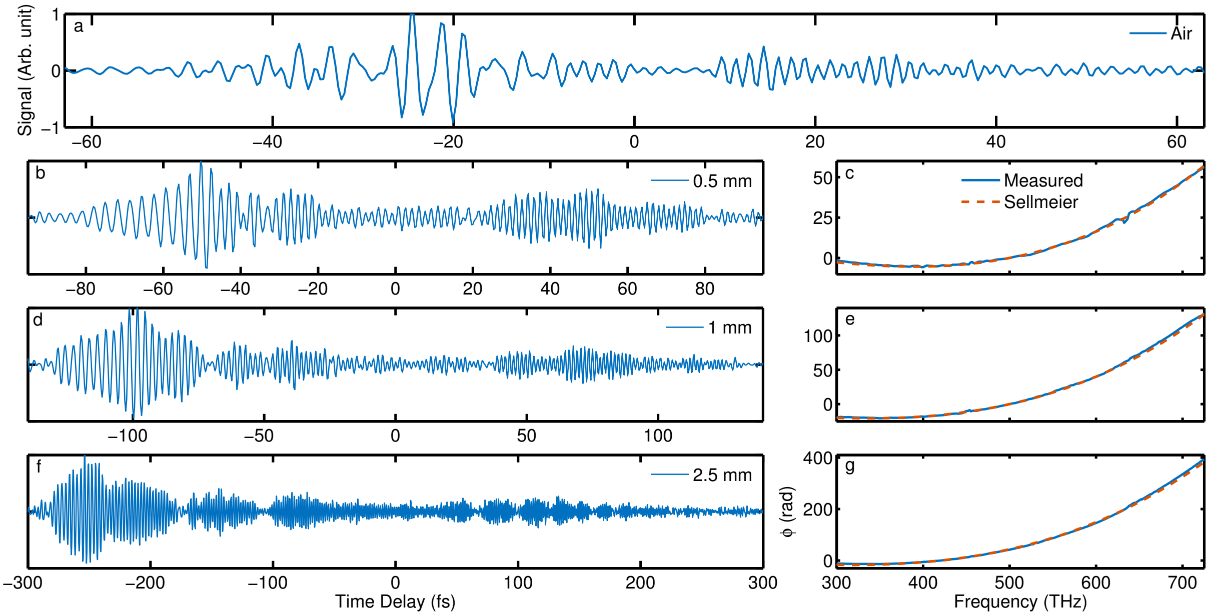

Supplementary Figure 14: NPS for dispersion measurement. The spectral phase accumulated by a broadband pulse sequence after propagating through a fused silica substrate is measured through measurements of waveforms with and without the sample. **a** Reference waveform consisting of a 750 nm pulse followed by its second harmonic. Waveforms are recorded after the pulse is dispersed in 0.5 mm, 1.0 mm, and 2.5 mm thick fused silica substrates, and are shown in **b**, **d**, and **f**, respectively. A comparison of the accumulated spectral phase obtained directly from the recorded waveforms with an analytical calculation from the Sellmeier equation are presented in **c**, **e**, and **g**, respectively

Supplementary References

[1] Keiber, S. *et al.* Electro-optic sampling of near-infrared waveforms. *Nature Photon.* **10**, 159-162 (2016).

[2] Bache, M. *et al.* The anisotropic Kerr nonlinear refractive index of the beta-barium borate (β - BaB_2O_4) nonlinear crystal. *Opt. Mat. Express* **3**, 357-382 (2013).

[3] Noda M., Yabana K. *et al.* SALMON: Scalable Ab-initio Light-Matter simulator for Optics and Nanoscience. *Computer Physics Communications* **235**, 356-365 (2018).

[4] Tran F. & Blaha, P. Accurate Band Gaps of Semiconductors and Insulators with a Semilocal Exchange-Correlation Potential. *Phys. Rev. Lett.* **102**, 226401 (2009).

[5] Wachter G. Simulation of condensed matter dynamics in strong femtosecond laser pulses. *PhD thesis*, Vienna University of Technology (2014).

[6] Zipp, L.J, Natan, A., and Bucksbaum, P. H. Probing electron delays in above-threshold ionization, *Optica* 6, 361-364 (2014).

[7] I. H. Malitson. Interspecimen comparison of the refractive index of fused silica, *J. Opt. Soc. Am.* **55**, 1205-1208 (1965).
